# Primary Mediastinal Hydatid Cyst Causing Diaphragmatic Palsy

**DOI:** 10.21470/1678-9741-2019-0001

**Published:** 2020

**Authors:** Madhusudhan M. Gopivallabha, Akshay Kumar Singh, Ashwini Kumar Pasarad, Ponnuswamy Parashuraman, Anil Kumar Govindareddy

**Affiliations:** 1 Sagar Hospitals Banashankari, DSI, Shavige Malleshwara Hill, Kumaraswamy Layout, Bengaluru, Karnataka, India.

**Keywords:** Pulmonary Echinococcosis, Mediastinal Cyst, Diaphragmatic Palsy, Recurrent Laryngeal Nerve

## Abstract

Hydatid cystic disease is a significant clinical problem in endemic countries. Hydatid cysts are most commonly located in the liver and lungs. Primary mediastinal hydatid cyst is a rare clinical entity. The diagnosis must be considered in a patient with a mediastinal mass, particularly in endemic regions. Mediastinal hydatid cysts causing paralysis of phrenic and recurrent laryngeal nerves have been rarely reported. We describe a rare case of primary mediastinal hydatid cyst associated with diaphragmatic palsy caused by compression of the left phrenic nerve, which was successfully treated with partial cystectomy and capitonnage with hemidiaphragmatic plication.

**Table:** 

Abbreviations, acronyms & symbols	
CT	= Computer tomography
MRI	= Magnetic resonance imaging

## INTRODUCTION

Echinococcosis is a significant clinical problem in endemic countries. Hydatid cysts are usually located in the liver, lungs, and less commonly, the brain. Primary mediastinal hydatid cysts are rare. It is difficult to diagnose a mediastinal hydatid cyst based on clinical findings alone. While many cases are diagnosed accidentally, some patients may present with related complications. Nevertheless, echinococcosis must be considered in all patients with a mediastinal mass in endemic regions. A thorough literature search showed that primary mediastinal hydatid cyst causing phrenic nerve compression leading to significant hemidiaphragmatic palsy has been rarely reported.

## CASE REPORT

**Fig. 1:**
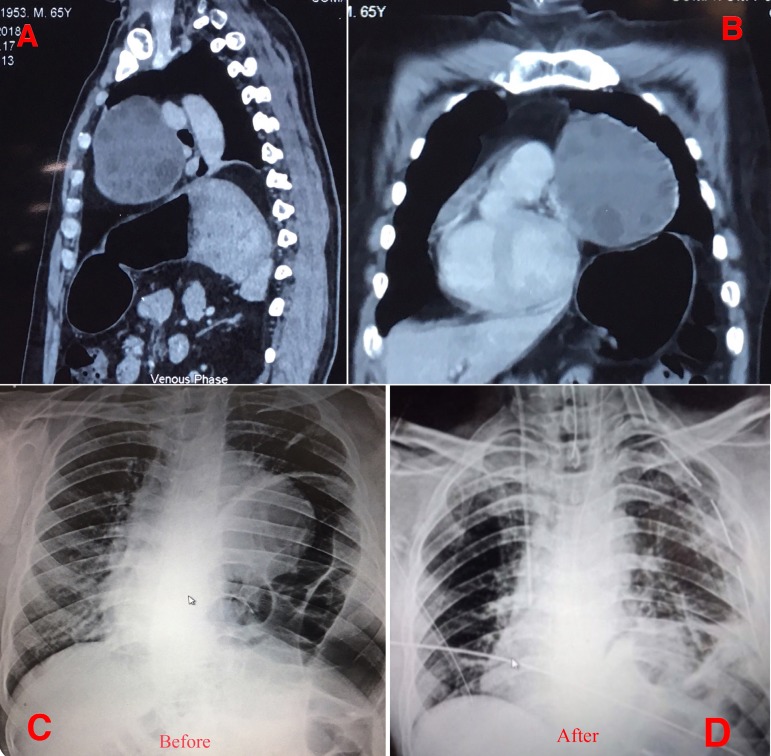
A and 1B - Computer tomography images showing the mediastinal cyst with daughter cysts. C and D - Pre and post left hemidiaphragmatic plication X-rays.

We present the case of a sixty-five-year-old male, who came to us with the complaint of gradually worsening dyspnoea. Clinical evaluation revealed decreased air entry over the left chest. Bowel sounds were audible over the left basal thorax. Thoracic computer tomography (CT) showed a mediastinal mass with features of hydatid cyst and an elevated left hemidiaphragm (Figure 1A and 1B).

**Fig. 2:**
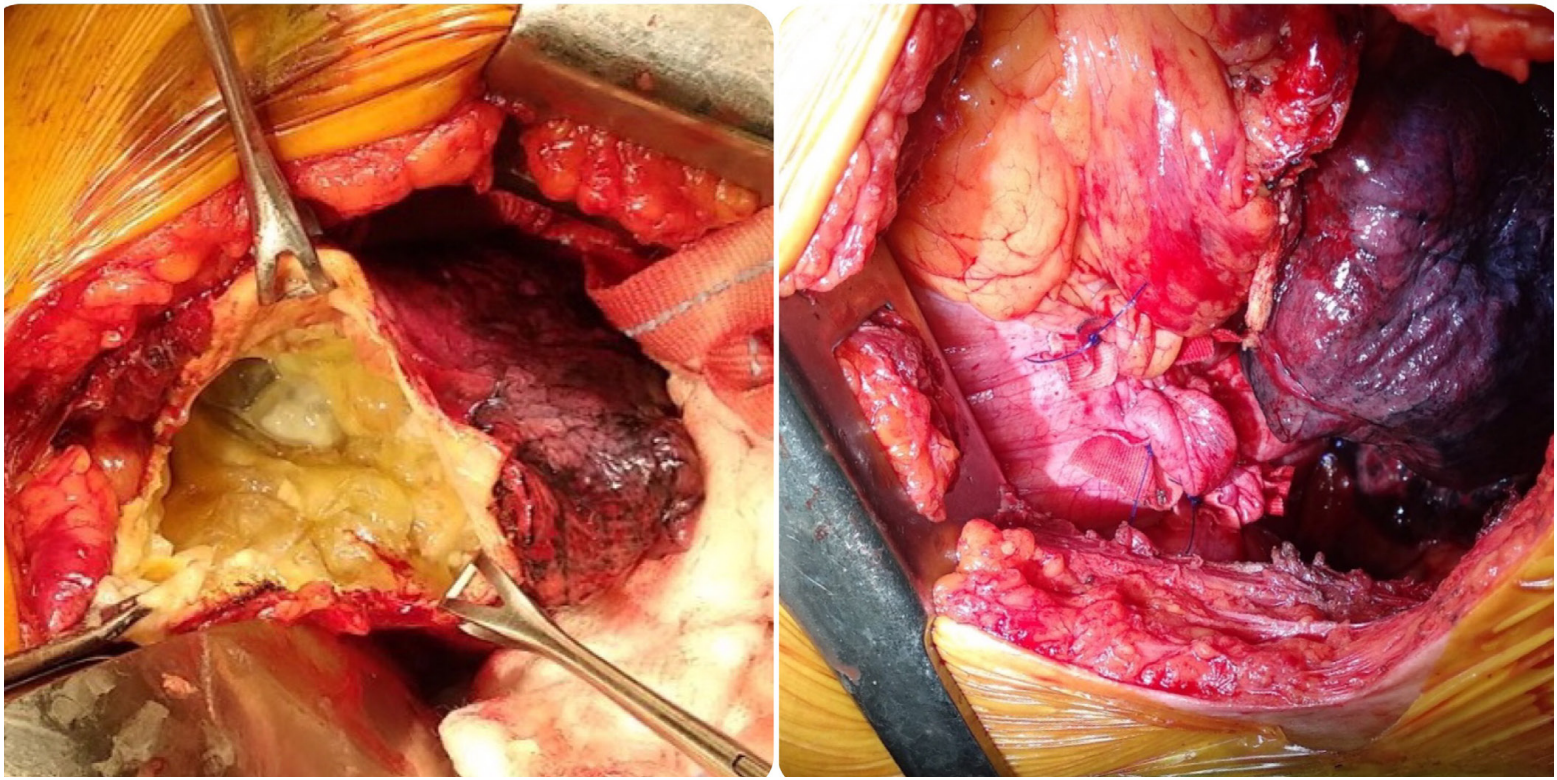
Left: hydatid cyst opened. Right: left hemidiaphragmatic plication.

Patient was taken up for surgery after preoperative workup. Left anterolateral thoracotomy was done. Pleura was entered through the left fourth intercostal space. A large cyst was noted, densely adherent to the lower lobe of the left lung and the pericardium. After packing the pleural cavity with mops soaked with hypertonic saline, cystotomy was done and cystic fluid was aspirated. Multiple daughter cysts were seen (Figure 2A); evacuated, avoiding spillage. The inner wall of the cyst was scraped, and the cavity washed with hypertonic saline. Partial cystectomy with capitonnage was done. Anteroposterior plication of left hemidiaphragm was done (Figure 2B). Wound was closed after placing an intercostal drainage tube.

Postoperative X-ray showed significant descent of the left hemidiaphragm (Figure 1C and 1D). Patient was treated with albendazole. He was discharged on the fourth postoperative day. Patient remains on follow-up.

## DISCUSSION

The term 'hydatid cyst' was coined by Rudolpti in 1800. The 'hydatid' (from the Greek, 'water drop') disease is caused by the parasite *Echinococcus granulosus* ^[^1^]^. The adult worm resides in the jejunum of the definitive host (dogs and other canines). It produces eggs that are passed in the stool. The eggs ingested by intermediate hosts (cows, sheep, humans) reach the duodenum and produce embryos, which enter the portal circulation. While most of these embryos get trapped in the liver, some of them pass through and reach other organs, developing into hydatid cysts^[2]^. The liver is the most common site for hydatid cysts, followed by the lungs. However, the hydatid cyst is known to occur in other unusual anatomical locations.

Cysts account for up to one-fourth of all mediastinal masses. Common mediastinal cysts include bronchogenic, thymic, and pleuropericardial cysts and lymphangiomas. Intrathoracic extrapulmonary hydatid cysts may be of mediastinal or pleural origin. Primary mediastinal hydatid cysts without pleuropulmonary connections are rare^[1]^. Rakower and Milwidsky, in their large series of more than 23,000 patients with hydatid disease, have reported only 0.1% of hydatid cysts in the mediastinal compartment and paravertebral sulcus^[3]^. In another large series of 1,619 cases of hydatid disease, Thameur and associates have reported 0.5% incidence of mediastinal hydatid cyst^[2]^.

Mediastinal echinococcosis is clinically indistinguishable from other mediastinal cysts. These cysts are usually discovered either incidentally or by complications like rupture. Symptoms and complications depend on size, location, and involvement of surrounding structures. Mediastinal hydatid cysts often present with atypical chest pain or signs of compression (cough, dyspnea, dysphagia, dysphonia)^[4]^. Occasionally, patients may present with non-specific symptoms. In one instance, a patient with hydatid cyst causing phrenic nerve compression was reported to have presented with just hiccups^[5]^.

Diagnosis can be obtained by correlating the clinical and radiological findings. Chest X-ray, CT, and magnetic resonance imaging (MRI) facilitate the diagnosis. CT is considered essential for displaying the morphology and defining the relationship of the lesion with the adjacent structures. Although a germinative membrane can sometimes be seen on an ultrasound or CT, the presence of calcification is very suspicious of a hydatid cyst. MRI may be particularly useful in the posterior mediastinal cysts (spinal involvement). Serological tests are often negative in intact/uncomplicated cysts. CT-guided fine-needle aspiration is considered dangerous because of the risk of rupture, dissemination, and anaphylactic shock^[6]^.

Various techniques like percutaneous drainage and capitonnage have been described to treat hydatid cysts. However, total excision of the cyst is considered the gold standard. It consists of a cystectomy along with total pericystectomy^[4]^. But when a hydatid cyst is closely related to vital mediastinal structures, total excision may not be possible, thereby allowing partial excision. Anti-helminthic drugs like albendazole and mebendazole are generally given during the postoperative period.

## CONCLUSION

Even though primary mediastinal hydatid cysts are rare, they should be considered in the differential diagnosis of all mediastinal cystic masses, particularly in the endemic regions. Surgery is the treatment of choice for relieving symptoms and preventing complications.

**Table:** 

Authors' roles & responsibilities	
MMG	Substantial contributions to the conception or design of the work; or the acquisition, analysis, or interpretation of data for the work; final approval of the version to be published
AKS	Substantial contributions to the conception or design of the work; or the acquisition, analysis, or interpretation of data for the work; final approval of the version to be published
AKP	Substantial contributions to the conception or design of the work; or the acquisition, analysis, or interpretation of data for the work; final approval of the version to be published
PP	Substantial contributions to the conception or design of the work; or the acquisition, analysis, or interpretation of data for the work; final approval of the version to be published
AKG	Substantial contributions to the conception or design of the work; or the acquisition, analysis, or interpretation of data for the work; final approval of the version to be published
